# Ultrathin-strut drug-eluting stents are associated with lower revascularization rates than drug-coated balloons in non-focal in-stent restenosis

**DOI:** 10.3389/fcvm.2026.1865693

**Published:** 2026-08-03

**Authors:** Berhan Keskin, Aykun Hakgor, Atakan Dursun, Aysel Akhundova, Umeyir Savur, Ebubekir Yigitbay, Ozlem Onder, Beytullah Cakal, Zubeyde Bayram, Bulent Demir, Haci Murat Gunes, Ibrahim Oguz Karaca, Ekrem Guler, Bilal Boztosun

**Affiliations:** Department of Cardiology, Istanbul Medipol University, Medipol Mega University Hospital, Istanbul, Türkiye

**Keywords:** drug-coated balloon, drug-eluting stent, in-stent restenosis, target-lesion revascularization, ultrathin-strut

## Abstract

**Background:**

In-stent restenosis (ISR) remains a significant clinical challenge despite advances in drug-eluting stent (DES) technology. Although current guidelines recommend drug-coated balloon (DCB) angioplasty or repeat DES implantation for DES-ISR, real-world data comparing DCB with newer-generation ultrathin-strut sirolimus-eluting stents (SES) are limited.

**Methods:**

This retrospective, single-center study included consecutive patients with stable angina who underwent treatment for DES-ISR using either a paclitaxel-coated DCB or an ultrathin-strut SES between January 2015 and May 2025. The primary outcome was target-lesion revascularization (TLR). Secondary endpoints included major adverse cardiac events (MACE), defined as a composite of all-cause mortality, myocardial infarction, stroke, and TLR. Time-to-event analyses were performed using Kaplan–Meier methods and Cox proportional hazards regression.

**Results:**

A total of 205 patients were included (DCB, *n* = 106; SES, *n* = 99), with a median follow-up of 863 days. In the overall cohort, TLR rates were comparable between groups. However, in the non-focal ISR subgroup, TLR was significantly higher in the DCB group compared with SES (34.1% vs. 12.7%, *p* = 0.024). Similarly, MACE rates were higher with DCB in non-focal ISR (39.0% vs. 18.2%, *p* = 0.041). No differences were observed in focal ISR. Multivariable analysis in the non-focal subgroup identified DCB treatment (HR 3.89, *p* = 0.006) as independent predictor of TLR. Kaplan–Meier analyses for TLR and MACE consistently demonstrated significantly worse outcomes with DCB in the non-focal ISR subgroup, whereas no significant differences were observed in focal ISR.

**Conclusions:**

Ultrathin-strut SES was associated with lower TLR and MACE rates compared with DCB in non-focal DES-ISR, whereas outcomes were comparable in focal ISR. These findings highlight the importance of lesion characteristics and support a tailored approach to ISR management.

## Introduction

Despite significant advancements in stent technology and the introduction of novel drug-eluting stents (DES), in-stent restenosis (ISR) remains a major clinical challenge in patients undergoing DES implantation. Although newer-generation DES are associated with lower ISR rates, approximately 6%–8% within the first year, they still carry an annual adverse event rate of around 2%, without evidence of reaching a plateau (1, 2). Current guidelines recommend either the use of a drug-coated balloon (DCB) or the implantation of an additional DES layer for the management of DES-ISR (3).

Among these, DCB therapy has emerged as a well-established alternative to repeat DES implantation in this patient population. Compared with conventional balloon angioplasty, DCB treatment has consistently demonstrated superior outcomes, including lower rates of target-lesion failure, target-lesion revascularization (TLR), and major adverse cardiac events (MACE) (4–6).

Several randomized trials and registries have subsequently compared DCB therapy with repeat DES implantation in patients with DES-ISR. Studies such as ISAR-DESIRE 3, PEPCAD China ISR, SEDUCE, DARE, and the analysis by Wanha et al. generally reported comparable rates of TLR, MACE, myocardial infarction, and mortality between DCB and DES strategies (7–12). However, other investigations have suggested a higher risk of recurrent restenosis and repeat revascularization with DCB during long-term follow-up (13–15). The largest evidence synthesis to date, the DAEDALUS study, demonstrated that repeat DES implantation was associated with lower rates of TLR than DCB treatment in patients with DES-ISR, highlighting the complexity of lesion selection and treatment decision-making in this population (14). Consequently, although DCB therapy has become widely adopted in contemporary practice, uncertainty remains regarding the optimal treatment strategy for specific DES-ISR lesion subsets.

Ultrathin-strut sirolimus-eluting stents (SES) have emerged as a newer generation of DES designed to reduce vessel injury, inflammatory response, and neointimal proliferation through lower metallic burden and improved vascular healing. However, evidence regarding their use in ISR lesions remains limited. The present study aims to compare the clinical outcomes of ultrathin-strut SES implantation vs. DCB treatment in patients with DES-ISR, thereby reflecting real-world practice and addressing the current paucity of data on the role of ultrathin-strut SES in this setting.

## Methods

### Study population

This retrospective, single-center study included consecutive patients with stable angina pectoris who underwent treatment with either a paclitaxel-coated DCB or an ultrathin-strut SES (Orsiro®, Biotronik AG, Bülach, Switzerland) for DES-ISR between January 1, 2015, and May 31, 2025. The exclusion criteria were as follows:

- End-stage renal failure (Glomerular filtration rate <30 mL/min/1.73 m^2^ or chronic hemodialysis) (*n* = 3)
- Acute coronary syndrome or cardiogenic shock (*n* = 13)
- Presence of more than moderate valvular disease (*n* = 3)
- Severe anemia (hemoglobin <8 mg/dL) (*n* = 2)
- Active malignancy (*n* = 1)
- Missing data (*n* = 14)
- Previous DES implantation for ISR (*n* = 4)
- Bail-out DES implantation following failed DCB application (*n* = 2)

**Figure 1:**
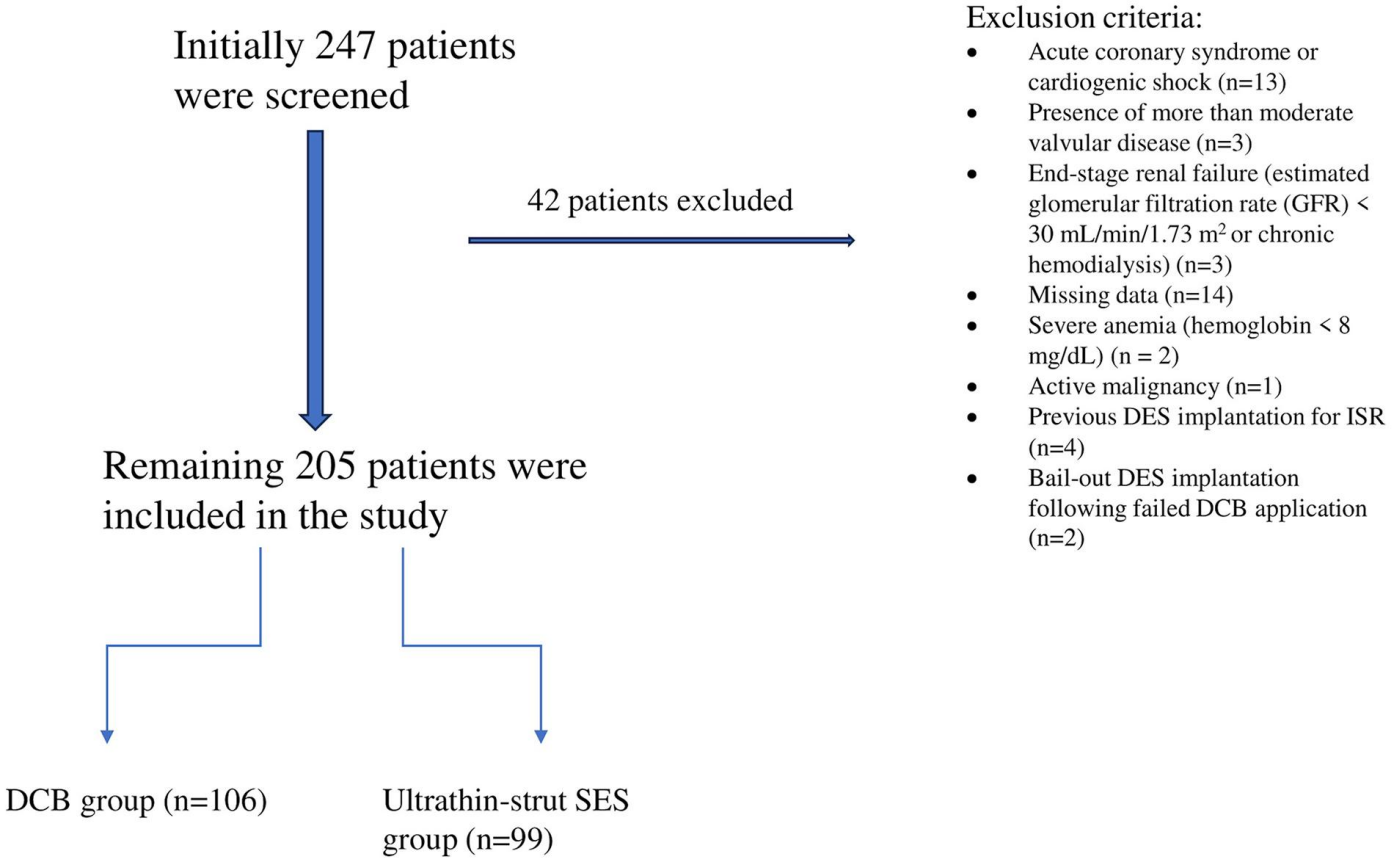
Study flow diagram.

After applying these exclusion criteria, the remaining 205 patients constituted the study population (Figure 1). All lesions included in the present study represented ISR occurring within previously implanted drug-eluting stents (DES-ISR). Patients with ISR developing within bare-metal stents were not included.

### Data collection and procedural details

Patient data on baseline clinical characteristics, comorbidities, medications, laboratory values, echocardiographic parameters, and angiographic findings were obtained from the hospital information system and patient files. Baseline characteristics, comorbidities, and laboratory values were recorded at the time of admission, while medication use during follow-up was also documented. Echocardiographic examinations were performed at discharge by a cardiac imaging specialist using a Vivid E95 ultrasound system (General Electric Vingmed Ultrasound, Milwaukee, WI) with a 3.5-MHz or M5S transducer. Routine echocardiographic parameters included left ventricular ejection fraction (assessed by visual estimation), systolic pulmonary artery pressure (PASP) calculated using the Bernoulli equation from tricuspid regurgitant jet velocity, and tricuspid annular plane systolic excursion (TAPSE) measured by M-mode.

All patients received a loading dose of clopidogrel (600 mg), aspirin (300 mg), and intravenous heparin according to body weight prior to percutaneous coronary intervention. All procedures were performed via radial arterial access. Pre-dilation with 1:1 sized plain balloons was performed in all cases, and non-compliant, scoring, or cutting balloons were used when necessary. Adequate lesion preparation was ensured before DCB or DES implantation in every patient. In the DCB group, drug-coated balloons were inflated for at least 60 s. Successful treatment was defined according to the Drug-Coated Balloon Academic Research Consortium criteria (15) as ≤30% residual stenosis, Thrombolysis in Myocardial Infarction (TIMI) grade 3 flow, and the absence of flow-limiting dissection. All patients underwent repeat angiographic assessment after a 5-minute waiting period to exclude acute recoil or flow-limiting dissection. Bailout DES implantation was performed in two cases of unsuccessful DCB treatment according to predefined criteria, and these patients were excluded from the study. In the ultrathin-strut SES group, routine post-dilation with a non-compliant balloon was performed in all patients. Intravascular ultrasound (IVUS) and optical coherence tomography (OCT) were not used in either group. All patients received at least 6 months of dual antiplatelet therapy (DAPT) with clopidogrel and aspirin. Continuation of DAPT beyond 6 months was left to the discretion of the treating physician according to individual patient and lesion characteristics.

Device selection (DCB vs. SES) was left to the discretion of the treating interventional cardiologists but followed consistent real-world criteria in line with contemporary ISR practice. A retrospective quantitative angiographic assessment was performed for study purposes. Lesion length was measured as the longitudinal extent of angiographic restenosis within the stented segment and/or within 5 mm proximal or distal to the stent edges. ISR morphology was subsequently classified according to the Mehran classification using these measurements.

This study was conducted in accordance with the Declaration of Helsinki, and approval was obtained from the institutional ethics committee (approval code: 951; date: July 31, 2025).

### Definitions

ISR was defined as a diameter stenosis >50% within the stented segment or within 5 mm of its proximal or distal margins, in accordance with the Drug-Coated Balloon Academic Research Consortium criteria (15). ISR lesions were further classified according to the Mehran criteria (16). Lesions were considered focal if they were ≤10 mm in length and located in the unscaffolded segment (i.e., articulation or gap), the body of the stent, the proximal or distal margin (but not both), or in multiple discrete sites (multifocal ISR). Diffuse ISR was defined as lesion length >10 mm. In addition, lesion severity was categorized as stable (≤90% stenosis), near-occlusion (>90% stenosis), or chronic total occlusion. The presence of bifurcation stenting at the lesion site was also recorded.

### Outcomes

The primary outcome of the study was TLR. TLR events were adjudicated retrospectively based on combined clinical and angiographic criteria. A TLR was recorded when a repeat PCI was performed within the previously treated segment (including 5 mm proximal or distal edges) in the presence of recurrent ischemic symptoms, objective evidence of ischemia, or angiographic restenosis ≥50%. Routine angiographic surveillance was not mandated. Follow-up coronary angiography was performed only when clinically indicated, including recurrent ischemic symptoms or objective evidence of myocardial ischemia. Therefore, TLR represented clinically driven repeat revascularization rather than angiographically detected restenosis identified through a protocol-mandated follow-up strategy. Secondary outcomes included major adverse cardiac events (MACE), long-term all-cause mortality, myocardial infarction, cerebrovascular accident, and significant bleeding events. MACE was defined as a composite of all-cause mortality, any myocardial infarction, any stroke, and target-lesion revascularization. Significant bleeding events were defined according to the Bleeding Academic Research Consortium (BARC) criteria as type 3–5 bleeding (17). Follow-up duration varied across patients because this was a retrospective real-world cohort without a standardized surveillance schedule. All patients who had available post–DES-ISR clinical follow-up after the index procedure were included in the survival analyses. Time-to-event outcomes were calculated from the date of the ISR intervention to the date of the event or last documented clinical contact.

### Statistical analysis

The distribution of variables was assessed using visual histograms and the Kolmogorov–Smirnov test. Normally distributed continuous variables were expressed as mean ± standard deviation, whereas non-normally distributed variables were presented as median and interquartile range (IQR; 25th–75th percentile). Categorical variables were expressed as counts (n) and percentages. Patients were stratified into two groups: the DCB group and the ultrathin-strut SES group.

Baseline clinical characteristics, comorbidities, medications, laboratory values, echocardiographic parameters, angiographic findings, and outcomes were compared between the two groups. Categorical variables were analyzed using the Chi-square (*χ*^2^) test or Fisher's exact test, as appropriate. Continuous variables were compared using the independent-samples *t*-test or the Mann–Whitney *U*-test, depending on data distribution. Differences in TLR and MACE during follow-up were evaluated using Kaplan–Meier cumulative event-free survival curves and the log-rank test. All comparisons were performed in the overall cohort and stratified according to focal and non-focal ISR subgroups.

Predictors of TLR were examined using stepwise univariate and multivariable Cox proportional hazards regression models in the overall cohort and non-focal ISR subgroup. Variables with *p* < 0.10 in univariate analysis were included in multivariable models. Hazard ratios (HRs) with 95% confidence intervals (CIs) were reported. Internal validation of the multivariable models was performed using bootstrap resampling with 1,000 iterations. Model performance was assessed by calculating the concordance index (C-index). Optimism was estimated as the mean difference between model performance in bootstrap samples and the original dataset, and an optimism-corrected C-index was derived to account for potential overfitting.

To further address confounding by indication, a propensity score-adjusted Cox proportional hazards analysis was performed. Propensity scores were estimated using logistic regression including age, sex, smoking status, diabetes mellitus, hypertension, chronic kidney disease, chronic obstructive pulmonary disease, atrial fibrillation, peripheral arterial disease, previous coronary artery bypass grafting, congestive heart failure, ISR morphology (non-focal vs. focal), bifurcation involvement, lesion severity (stable lesion, chronic total occlusion, and near-occlusion), left main coronary artery (LMCA) stenting, and SYNTAX score. The estimated propensity score was subsequently entered as an adjustment covariate in Cox proportional hazards models evaluating TLR. The proportional hazards assumption was assessed using Schoenfeld residuals for all Cox proportional hazards models.

To evaluate whether the effect of treatment strategy differed according to ISR morphology, a formal interaction analysis was performed. Cox proportional hazards models were constructed including treatment modality (ultrathin-strut SES vs. DCB), ISR morphology (non-focal vs. focal), and a treatment-by-morphology interaction term. To further account for potential confounding by indication, a propensity score-adjusted interaction analysis was performed as a sensitivity analysis using the propensity score model described above. The estimated propensity score was entered as an adjustment covariate in the interaction model. A statistically significant interaction term was interpreted as evidence that the comparative treatment effect differed according to ISR morphology.

A two-tailed *p*-value <0.05 was considered statistically significant. All statistical analyses were performed using Python, version 3.14.0 (Python Software Foundation, Wilmington, DE, USA).

## Results

A total of 205 patients were included in the study, of whom 106 were treated with DCB and 99 with ultrathin-strut SES. Based on ISR morphology, 96 patients had non-focal ISR (DCB: *n* = 41; SES: *n* = 55) and 109 had focal ISR (DCB: *n* = 65; SES: *n* = 44).

In the overall cohort, patients treated with DCB were younger than those treated with SES (58.1 ± 9.3 vs. 61.6 ± 10.4 years, *p* = 0.009), although the absolute difference was modest. This difference remained significant in the non-focal ISR subgroup (56.4 ± 8.9 vs. 61.1 ± 11.2 years, *p* = 0.037), whereas it was not statistically significant in the focal ISR subgroup (*p* = 0.074) (Table 1). Other baseline clinical characteristics were well balanced between the treatment groups. There were no significant differences in sex distribution, smoking status, or major comorbidities including hypertension, diabetes mellitus, chronic kidney disease, chronic obstructive pulmonary disease, atrial fibrillation, prior stroke, heart failure, peripheral arterial disease, or previous CABG in the overall cohort or within ISR subgroups (Table 1).

**Table 1:** Comparison of baseline characteristics, comorbidities, and medications between drug-coated balloon and ultrathin-strut sirolimus-eluting stent groups.

Variables	Overall cohort			Non-focal ISR			Focal ISR		
	DCB (*n* = 106)	SES (*n* = 99)	*p*-value	DCB (*n* = 41)	SES (*n* = 55)	*p*-value	DCB (*n* = 65)	SES (*n* = 44)	*p*-value
Baseline Clinical Characteristics & Comorbidities									
Age (years)	58.11 ± 9.33	61.61 ± 10.38	0.009	56.41 ± 8.89	61.11 ± 11.23	0.037	59.18 ± 9.51	62.23 ± 9.30	0.074
Male sex	88 (83.0%)	81 (81.8%)	0.966	35 (85.4%)	47 (85.5%)	1.000	53 (81.5%)	34 (77.3%)	0.763
Smoking	51 (48.1%)	56 (56.6%)	0.284	22 (53.7%)	35 (63.6%)	0.439	29 (44.6%)	21 (47.7%)	0.901
Hypertension	89 (84.0%)	75 (75.8%)	0.196	33 (80.5%)	38 (69.1%)	0.306	56 (86.2%)	37 (84.1%)	0.982
Diabetes mellitus	57 (53.8%)	46 (46.5%)	0.365	22 (53.7%)	23 (41.8%)	0.346	35 (53.8%)	23 (52.3%)	1.000
Chronic kidney disease	4 (3.8%)	7 (7.1%)	0.461	2 (4.9%)	5 (9.1%)	0.695	2 (3.1%)	2 (4.5%)	1.000
Chronic obstructive pulmonary disease	6 (5.7%)	2 (2.0%)	0.325	4 (9.8%)	2 (3.6%)	0.397	2 (3.1%)	0 (0.0%)	0.514
Atrial fibrillation	11 (10.4%)	7 (7.1%)	0.556	4 (9.8%)	2 (3.6%)	0.397	7 (10.8%)	5 (11.4%)	1.000
Previous stroke	4 (3.8%)	2 (2.0%)	0.742	1 (2.4%)	0 (0.0%)	0.427	3 (4.6%)	2 (4.5%)	1.000
Congestive heart failure	12 (11.3%)	10 (10.1%)	0.955	5 (12.2%)	6 (10.9%)	1.000	7 (10.8%)	4 (9.1%)	1.000
Peripheral arterial Disease	8 (7.5%)	3 (3.0%)	0.261	4 (9.8%)	2 (3.6%)	0.397	4 (6.2%)	1 (2.3%)	0.646
Previous CABG	12 (11.3%)	15 (15.2%)	0.546	4 (9.8%)	10 (18.2%)	0.387	8 (12.3%)	5 (11.4%)	1.000
Medications During Follow-up									
Statin usage	103 (97.2%)	99 (100.0%)	0.269	41 (100.0%)	55 (100.0%)	1.000	62 (95.4%)	44 (100.0%)	0.271
ß-blocker usage	98 (92.5%)	92 (92.9%)	1.000	37 (90.2%)	52 (94.5%)	0.456	61 (93.8%)	40 (90.9%)	0.712
ACEi/ARB usage	80 (75.5%)	70 (70.7%)	0.541	28 (68.3%)	36 (65.5%)	0.942	52 (80.0%)	34 (77.3%)	0.918
SGLT-2i usage	23 (21.7%)	15 (15.2%)	0.305	9 (22.0%)	8 (14.5%)	0.503	14 (21.5%)	7 (15.9%)	0.629
MRA usage	8 (7.5%)	4 (4.0%)	0.441	4 (9.8%)	3 (5.5%)	0.456	4 (6.2%)	1 (2.3%)	0.646
ARNI usage	4 (3.8%)	3 (3.0%)	1.000	3 (7.3%)	1 (1.8%)	0.310	1 (1.5%)	2 (4.5%)	0.564
Insulin usage	12 (11.3%)	9 (9.1%)	0.767	5 (12.2%)	4 (7.3%)	0.490	7 (10.8%)	5 (11.4%)	1.000
Anticoagulation	11 (10.4%)	5 (5.1%)	0.246	4 (9.8%)	2 (3.6%)	0.397	7 (10.8%)	3 (6.8%)	0.737
DAPT_duration (months)	12.00 (9.25–66.00)	20.00 (11.00–50.00)	0.248	12.00 (11.00–85.00)	38.00 (12.00–52.50)	0.339	12.00 (8.00–61.00)	17.00 (8.00–40.50)	0.687

DCB, Drug-coated balloon; SES, Ultrathin-strut Sirolimus-Eluting stent; CABG, Coronary artery bypass graft surgery; ACEi, Angiotensin converting enzyme inhibitors; ARB, Angiotensin II receptor blocker; SGLT-2i, Sodium-glucose cotransporter-2 inhibitors; MRA, Mineralocorticoid receptor antagonist; ARNI, Angiotensin receptor neprilysin inhibitor; DAPT, Dual anti-platelet therapy.

Medication use during follow-up was comparable between groups. The use of statins, beta-blockers, ACE inhibitors/ARBs, SGLT-2 inhibitors, mineralocorticoid receptor antagonists, angiotensin receptor–neprilysin inhibitors, insulin, and oral anticoagulation did not differ significantly between DCB and SES groups across the overall population and subgroup analyses (Table 1). Median dual antiplatelet therapy (DAPT) durations were also comparable between groups (Table 1). Overall, apart from younger age in the DCB group, baseline characteristics and medical therapy were well balanced between treatment strategies.

In the overall cohort, laboratory parameters were largely comparable between the DCB and SES groups, with no significant differences across hematological, renal, metabolic, inflammatory, or lipid profiles (Table 2). These findings were consistent in both non-focal and focal ISR subgroups. Echocardiographic parameters, including LVEF, PASP, and TAPSE, were also similar between groups in the overall population and subgroup analyses (Table 2). Finally, median follow-up duration did not differ significantly between treatment groups in the overall cohort [709 [537.8–2,100.0] vs. 879 [528.5–1,730.5] days, *p* = 0.458] or within non-focal and focal ISR subgroups (all *p* > 0.05), indicating comparable follow-up times across groups (Table 2). The median follow-up duration in the overall cohort was 863 [527–1773] days.

**Table 2:** Comparison of laboratory values, echocardiographic parameters, and follow-up durations between drug-coated balloon and ultrathin-strut sirolimus-eluting stent groups.

Variables	Overall cohort			Non-focal ISR			Focal ISR		
	DCB (*n* = 106)	SES (*n* = 99)	*p*-value	DCB (*n* = 41)	SES (*n* = 55)	*p*-value	DCB (*n* = 65)	SES (*n* = 44)	*p*-value
Laboratory Values									
Hemoglobin (g/dL)	13.61 ± 1.75	13.63 ± 1.66	0.957	13.79 ± 1.89	13.52 ± 1.72	0.415	13.50 ± 1.66	13.76 ± 1.58	0.411
WBC (cell count/L)	8.33 ± 2.29	8.19 ± 1.95	0.891	8.36 ± 2.27	8.11 ± 1.92	0.670	8.31 ± 2.32	8.30 ± 2.01	0.802
Platelets (cell count/mcL)	243.68 ± 81.8	248.63 ± 79.8	0.697	236.56 ± 91.73	242.62 ± 68.28	0.586	248.17 ± 75.38	256.14 ± 92.55	0.597
Creatinine (mg/dL)	0.89 (0.78–1.00)	0.89 (0.78–1.00)	0.746	0.89 (0.78–0.99)	0.91 (0.83–1.00)	0.493	0.87 (0.77–1.00)	0.82 (0.77–0.97)	0.557
Urea (mg/dL)	33.58 ± 15.68	35.30 ± 22.25	0.524	38.34 ± 20.29	36.46 ± 25.29	0.294	30.92 ± 11.94	34.19 ± 19.57	0.933
AST (IU/L)	26.99 ± 16.12	22.00 ± 10.0	0.102	29.94 ± 20.47	22.17 ± 12.79	0.103	25.24 ± 12.87	21.82 ± 6.23	0.497
Albumin (g/dL)	4.49 ± 0.49	4.17 ± 0.49	0.113	4.60 ± 0.39	4.14 ± 0.46	0.111	4.42 ± 0.55	4.21 ± 0.55	0.713
Sodium (mEq/L)	137.86 ± 3.45	138.68 ± 3.2	0.128	137.96 ± 4.19	138.13 ± 3.52	0.667	137.81 ± 2.94	139.29 ± 2.88	0.068
Potassium (mEq/L)	4.24 ± 0.46	4.32 ± 0.59	0.306	4.31 ± 0.44	4.36 ± 0.57	0.593	4.21 ± 0.48	4.27 ± 0.62	0.612
CRP (mg/dL)	3.24 (1.90–9.40)	2.26 (1.01–4.35)	0.151	2.97 (1.95–15.71)	2.50 (1.11–4.67)	0.476	3.76 (1.53–6.46)	2.12 (0.73–3.79)	0.134
Total cholesterol (mg/dL)	177.84 ± 58.5	175.30 ± 54.	0.744	185.19 ± 72.09	178.61 ± 66.13	0.677	171.42 ± 45.08	171.23 ± 38.47	0.807
LDL-cholesterol (mg/dL)	103.59 ± 40.3	102.12 ± 39.9	0.895	108.19 ± 45.89	101.62 ± 41.89	0.430	100.29 ± 35.78	102.77 ± 37.54	0.588
Triglycerides (mg/dL)	180.00 (109.20–283.00)	152.00 (122.00–202.00)	0.404	169.00 (119.10–244.30)	155.50 (137.00–186.25)	0.722	209.00 (103.75–290.00)	151.00 (99.00–290.00)	0.656
HDL-cholesterol (mg/dL)	45.19 ± 15.34	44.78 ± 11.5	0.850	40.30 ± 8.91	43.41 ± 12.92	0.664	49.05 ± 18.27	46.58 ± 9.57	0.969
Hemoglobin A1c (%)	6.94 ± 1.41	6.92 ± 1.65	0.972	6.80 ± 1.54	7.13 ± 2.23	0.592	7.04 ± 1.33	6.73 ± 0.95	0.618
Echocardiographic Parameters									
Left ventricular ejection fraction (%)	55.25 ± 10.02	56.12 ± 9.67	0.528	54.58 ± 9.29	55.57 ± 10.25	0.370	55.67 ± 10.51	56.83 ± 8.93	0.801
Pulmonary artery systolic pressure (mmHg)	29.19 ± 8.15	28.76 ± 7.57	0.922	27.50 ± 5.89	28.40 ± 9.16	0.927	30.18 ± 9.25	29.29 ± 4.75	0.805
TAPSE (cm)	2.04 ± 0.53	2.20 ± 0.30	0.615	2.50 ± 0.14	2.16 ± 0.34	0.194	1.88 ± 0.52	2.33 ± 0.10	0.130
Follow-up Duration (days)	709.00 (537.75–2,100.00)	879.00 (528.50–1,730.50)	0.458	695.00 (570.00–2,355.00)	1,435.00 (637.00–1,867.50)	0.243	801.00 (503.00–1,945.00)	790.00 (499.75–1,487.00)	0.654

DCB, Drug-coated balloon; SES, Ultrathin-strut Sirolimus-Eluting stent; WBC, White blood cell; AST, Aspartate aminotransferase; ALT, Alanine aminotransferase; CRP, C-reactive protein; LDL, Low-density lipoprotein; HDL, High-density lipoprotein; TSH, Thyroid-stimulating hormone; TAPSE, Tricuspid annular plane systolic excursion.

In the overall cohort, angiographic characteristics were largely comparable between DCB and SES groups, including the number of diseased vessels and SYNTAX I scores, as well as the presence of LMCA stenting (Table 3). Device length was significantly shorter in the DCB group compared with the SES group (25.0 ± 6.0 mm vs. 35.3 ± 15.7 mm, *p* < 0.001), a difference that was consistent across both non-focal and focal ISR subgroups (all *p* < 0.001) (Table 3). Lesion distribution by vessel and bifurcation involvement did not differ between groups. In the overall cohort, diffuse lesions were less frequent in the DCB group (30.2% vs. 44.4%, *p* = 0.049), whereas focal lesions were more common (61.3% vs. 44.4%, *p* = 0.023) (Table 3). Regarding lesion severity, stable lesions (≤90% stenosis) were more frequent in the DCB group compared with the SES group (84.0% vs. 66.7%, *p* = 0.007), indicating a higher burden of severe lesions in the SES group; this difference remained evident in the non-focal ISR subgroup (87.8% vs. 67.3%, *p* = 0.037), whereas lesion severity was comparable in the focal ISR subgroup (Table 3).

**Table 3:** Comparison of angiographic characteristics and outcomes between drug-coated balloon and ultrathin-strut sirolimus-eluting stent groups.

Variables	Overall cohort			Non-focal ISR			Focal ISR		
	DCB (*n* = 106)	SES (*n* = 99)	*p*-value	DCB (*n* = 41)	SES (*n* = 55)	*p*-value	DCB (*n* = 65)	SES (*n* = 44)	*p*-value
Angiographic Characteristics									
Diseased vessel number	2.25 ± 0.71	2.36 ± 0.71	0.214	2.32 ± 0.72	2.35 ± 0.73	0.830	2.20 ± 0.71	2.39 ± 0.69	0.168
SYNTAX I score	10.91 ± 7.57	11.62 ± 8.41	0.758	10.61 ± 6.48	14.01 ± 8.81	0.065	11.11 ± 8.26	8.81 ± 7.03	0.219
Device length (mm)	25.02 ± 5.96	35.30 ± 15.7	<0.001	27.90 ± 5.76	38.89 ± 17.87	<0.001	23.20 ± 5.38	30.82 ± 11.06	<0.001
Device diameter (mm)	3.12 ± 0.41	3.00 ± 0.39	0.074	3.06 ± 0.33	3.02 ± 0.32	0.584	3.12 ± 0.41	2.94 ± 0.40	0.036
Presence of LMCA stent	24 (22.6%)	19 (19.2%)	0.664	8 (19.5%)	9 (16.4%)	0.897	16 (24.6%)	10 (22.7%)	1.000
Lesion involving bifurcation stent	14 (13.2%)	7 (7.1%)	0.223	5 (12.2%)	3 (5.5%)	0.281	9 (13.8%)	4 (9.1%)	0.652
Lesion site			0.714			0.872			0.365
LAD	53 (50.0%)	41 (41.4%)		25 (61.0%)	30 (54.5%)		28 (43.1%)	11 (25.0%)	
CX or OM	21 (19.8%)	21 (21.2%)		5 (12.2%)	5 (9.1%)		16 (24.6%)	16 (36.4%)	
RCA	26 (24.5%)	32 (32.3%)		9 (22.0%)	17 (30.9%)		17 (26.2%)	15 (34.1%)	
DX	3 (2.8%)	2 (2.0%)		1 (2.4%)	1 (1.8%)		2 (3.1%)	1 (2.3%)	
SVG	3 (2.8%)	3 (3.0%)		1 (2.4%)	2 (3.6%)		2 (3.1%)	1 (2.3%)	
Lesion type						1.000			
Diffuse	32 (30.2%)	44 (44.4%)	0.049	32 (78.0%)	44 (80.0%)		N/A	N/A	N/A
Focal	65 (61.3%)		0.023						
Multifocal	9 (8.5%)	44 (44.4%)	0.692	N/A	N/A				
		11 (11.1%)		9 (22.0%)	11 (20.0%)				
Lesion severity									
Chronic total occlusion	0 (0.0%)	5 (5.1%)	0.059	0 (0.0%)	5 (9.1%)	0.069	0 (0.0%)	0 (0.0%)	1.000
Near-occlusion (>90% stenosis)	17 (16.0%)	28 (28.3%)	0.051	5 (12.2%)	13 (23.6%)	0.248	12 (18.5%)	15 (34.1%)	0.103
Stable (≤ 90% stenosis)	89 (84.0%)	66 (66.7%)	0.007	36 (87.8%)	37 (67.3%)	0.037	53 (81.5%)	29 (65.9%)	0.103
Outcomes									
Procedural mortality	0 (0.0%)	0 (0.0%)	1.000	0 (0.0%)	0 (0.0%)	1.000	0 (0.0%)	0 (0.0%)	1.000
Major procedural complication	0 (0.0%)	0 (0.0%)	1.000	0 (0.0%)	0 (0.0%)	1.000	0 (0.0%)	0 (0.0%)	1.000
30-day mortality	0 (0.0%)	1 (1.0%)	0.483	0 (0.0%)	0 (0.0%)	1.000	0 (0.0%)	1 (2.3%)	0.404
Target-lesion revascularization	28 (26.4%)	17 (17.2%)	0.153	14 (34.1%)	7 (12.7%)	0.024	14 (21.5%)	10 (22.7%)	1.000
Long-term mortality	8 (7.5%)	6 (6.1%)	0.885	2 (4.9%)	4 (7.3%)	1.000	6 (9.2%)	2 (4.5%)	0.470
Myocardial infarction	1 (0.9%)	1 (1.0%)	1.000	1 (2.4%)	0 (0.0%)	0.427	0 (0.0%)	1 (2.3%)	0.404
Stroke	1 (0.9%)	0 (0.0%)	1.000	0 (0.0%)	0 (0.0%)	1.000	1 (1.5%)	0 (0.0%)	1.000
MACE	37 (34.9%)	22 (22.2%)	0.064	16 (39.0%)	10 (18.2%)	0.041	21 (32.3%)	12 (27.3%)	0.727
BARC type 3–5 bleeding	1 (0.9%)	1 (1.0%)	1.000	1 (2.4%)	0 (0.0%)	0.427	0 (0.0%)	1 (2.3%)	0.404

DCB, Drug-coated balloon; SES, Ultrathin-strut Sirolimus-Eluting stent; LMCA, Left main coronary artery; LAD, Left anterior descending artery; CX, Circumflex artery; OM, Obtuse marginal artery; RCA, Right coronary artery; DX, Diagonal artery; SVG, Saphenous vein grafts; MACE, Major adverse cardiac events; BARC, Bleeding Academic Research Consortium.

No procedural mortality or major procedural complications, including vessel rupture, flow-limiting dissection, or cardiac tamponade, were observed in either treatment group (Table 3). Thirty-day mortality was comparable between groups. The primary outcome of the study, TLR, was comparable between groups in the overall cohort (26.4% vs. 17.2%, *p* = 0.153) (Table 3). In subgroup analyses, TLR was significantly higher in the DCB group in the non-focal ISR subgroup (34.1% vs. 12.7%, *p* = 0.024), while similar rates were observed in the focal ISR subgroup (21.5% vs. 22.7%, *p* = 1.000). Long-term all-cause mortality, myocardial infarction, stroke, and BARC type 3–5 bleeding events were comparable between groups (Table 3). The incidence of MACE was numerically higher in the DCB group in the overall cohort (34.9% vs. 22.2%, *p* = 0.064) and was significantly higher in the non-focal ISR subgroup (39.0% vs. 18.2%, *p* = 0.041), whereas no difference was observed in the focal ISR subgroup (32.3% vs. 27.3%, *p* = 0.727) (Table 3).

**Figure 2:**
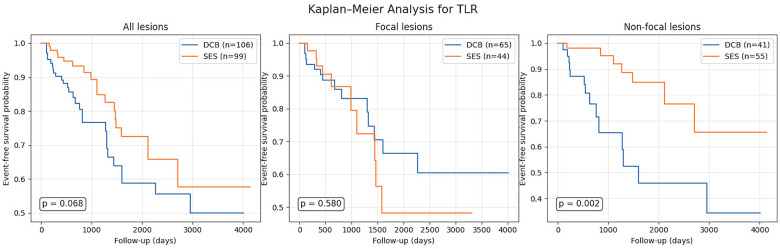
Kaplan–meier curves for target-lesion revascularization (TLR).

**Figure 3:**
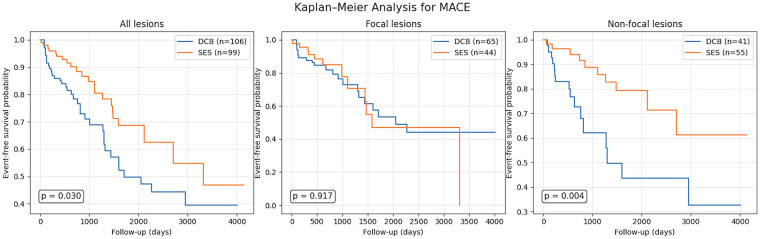
Kaplan–meier curves for major adverse cardiac events (MACE).

Kaplan–Meier analysis for the primary endpoint of TLR showed no statistically significant difference between DCB and SES in the overall cohort (log-rank *p* = 0.068), although there was a numerical trend toward higher TLR rates in the DCB group (Figure 2). In subgroup analyses, TLR rates were similar between treatment strategies in focal ISR (log-rank *p* = 0.580), whereas significantly higher TLR rates were observed in the DCB group in patients with non-focal ISR (log-rank *p* = 0.002) (Figure 2). For MACE, Kaplan–Meier analysis demonstrated a significantly higher event rate in the DCB group in the overall cohort (log-rank *p* = 0.030) (Figure 3). This difference was driven by the non-focal ISR subgroup, where MACE rates were significantly higher in the DCB group (log-rank *p* = 0.004), while no difference was observed in patients with focal ISR (log-rank *p* = 0.917) (Figure 3). Overall, time-to-event analyses were consistent with crude event comparisons, indicating that the unfavorable outcomes associated with DCB were primarily confined to patients with non-focal ISR, whereas comparable results were observed between treatment strategies in focal ISR.

**Figure 4:**
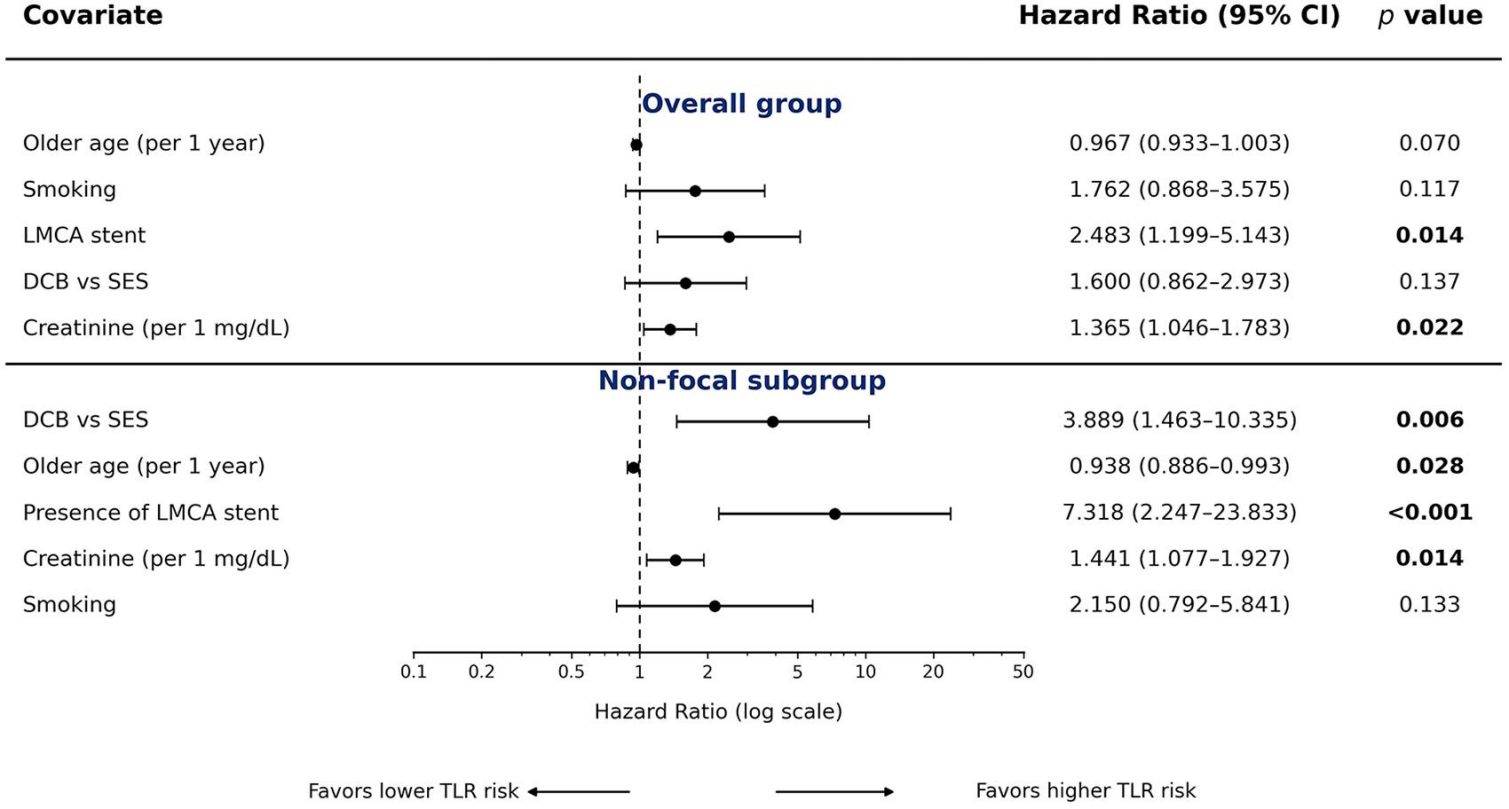
Forest plot of multivariable Cox regression analyses for TLR in the overall cohort and non-focal ISR subgroup.

In the overall cohort, there were 45 TLR events during follow-up. Univariate Cox regression analysis showed that age (HR 0.957, 95% CI 0.927–0.987, *p* = 0.006), smoking (HR 2.174, 95% CI 1.153–4.098, *p* = 0.016), presence of LMCA stenting (HR 2.287, 95% CI 1.125–4.647, *p* = 0.022), creatinine level (HR 1.316, 95% CI 0.973–1.781, *p* = 0.075), and treatment strategy (DCB vs. SES) (HR 1.747, 95% CI 0.953–3.201, *p* = 0.071) were associated with TLR (Table 4). In multivariable analysis, creatinine level (HR 1.365, 95% CI 1.046–1.783, *p* = 0.022) and presence of LMCA stenting (HR 2.483, 95% CI 1.199–5.143, *p* = 0.014) emerged as independent predictors of TLR (Table 4, Figure 4). Treatment strategy (DCB vs. SES) was not an independent predictor of TLR (HR 1.600, 95% CI 0.862–2.973, *p* = 0.137). Bootstrap internal validation (1,000 resamples) demonstrated limited optimism (C-index optimism 0.038) with preservation of discrimination after correction (optimism-corrected C-index 0.64), indicating modest but acceptable model performance.

**Table 4:** Univariate and multivariable Cox regression analysis for predicting target-lesion revascularization in the overall cohort.

**Univariate Cox Regression**				**Multivariable Cox Regression**		
**Predictor**	**Hazard Ratio**	**95% Confidence Interval**	***p*-value**	**Hazard Ratio**	**95% Confidence Interval**	***p*-value**
DCB vs. SES	1.747	0.953–3.201	**0** **.** **071**	1.600	0.862–2.973	0.137
Age (per 1 year)	0.957	0.927–0.987	**0** **.** **006**	0.967	0.933–1.003	0.070
Male sex	1.948	0.821–4.620	0.130			
Smoking	2.174	1.153–4.098	**0** **.** **016**	1.762	0.868–3.575	0.117
Diabetes mellitus	1.526	0.847–2.748	0.159			
Hypertension	0.699	0.352–1.390	0.308			
Previous CABG	1.195	0.556–2.571	0.648			
Atrial fibrillation	1.152	0.411–3.230	0.788			
Pre-existing COPD	0.618	0.085–4.511	0.635			
Congestive heart failure	0.557	0.173–1.797	0.327			
Hemoglobin (per 1 g/dL)	0.97	0.82–1.15	0.726			
Creatinine (per 1 mg/dL)	1.316	0.973–1.781	**0** **.** **075**	1.365	1.046–1.783	**0** **.** **022**
CRP (per 1 mg/dL)	1.004	0.987–1.022	0.617			
ß-blocker usage	1.374	0.332–5.680	0.661			
Insulin usage	1.741	0.684–4.434	0.245			
Anticoagulation	1.267	0.452–3.550	0.652			
ACEi/ARB usage	0.916	0.472–1.779	0.796			
SGLT-2i usage	0.943	0.398–2.234	0.893			
Left ventricular ejection fraction (per 1%)	1.010	0.978–1.042	0.550			
PASP (per 1 mmHg)	0.959	0.876–1.050	0.363			
SYNTAX I score (per 1 unit)	1.005	0.964–1.048	0.821			
Presence of LMCA stent	2.287	1.125–4.647	**0** **.** **022**	2.483	1.199–5.143	**0** **.** **014**
Lesion type (Non-focal vs. Focal)	0.825	0.459–1.486	0.522			
Lesion severity Stable vs. near-occlusion/CTO	0.640	0.347–1.181	0.153			
Device diameter (per 1 mm)	1.694	0.791–3.629	0.175			
Device length (per 1 mm)	1.000	0.977–1.023	0.976			

CABG, Coronary artery bypass graft surgery; COPD,: Chronic obstructive pulmonary disease; CRP, C-reactive protein;, ACEi, Angiotensin converting enzyme inhibitors; ARB, Angiotensin II receptor blocker; SGLT-2i, Sodium-glucose cotransporter-2 inhibitors; PASP, Pulmonary artery systolic pressure; LMCA, Left main coronary artery; CTO, Chronic total occlusion; DCB, Drug-coated balloon; SES, Ultrathin-strut sirolimus-eluting stent.

Bold values indicate statistical significance (*p* < 0.05).

In the non-focal ISR subgroup, 21 TLR events occurred during follow-up. Univariate Cox regression analysis showed that age (HR 0.936, 95% CI 0.894–0.979, *p* = 0.004), presence of LMCA stenting (HR 4.682, 95% CI 1.573–13.93, *p* = 0.006), treatment strategy (DCB vs. SES) (HR 3.972, 95% CI 1.585–9.954, *p* = 0.003), smoking (HR 2.353, 95% CI 0.900–6.150, *p* = 0.081), and creatinine level (HR 1.338, 95% CI 0.971–1.844, *p* = 0.075) were associated with TLR (Table 5). In multivariable analysis, older age remained independently associated with a lower risk of TLR (HR 0.938, 95% CI 0.886–0.993, *p* = 0.028), while creatinine level (HR 1.441, 95% CI 1.077–1.927, *p* = 0.014), presence of LMCA stenting (HR 7.318, 95% CI 2.247–23.83, *p* < 0.001), and treatment strategy (DCB vs. SES) (HR 3.889, 95% CI 1.463–10.335, *p* = 0.006) emerged as independent predictors of TLR (Table 5, Figure 4). Bootstrap internal validation (1,000 resamples) showed low optimism and consistent model performance after correction, supporting the internal validity of the multivariable model. The optimism-corrected C-index was 0.77, indicating good discriminative ability.

**Table 5:** Univariate and multivariable Cox regression analysis for predicting target-lesion revascularization in the Non-focal ISR.

**Univariate Cox Regression**				**Multivariable Cox Regression**		
**Predictor**	**Hazard Ratio**	**95% Confidence Interval**	***p*-value**	**Hazard Ratio**	**95% Confidence Interval**	***p*-value**
DCB vs. SES	3.972	1.585–9.954	**0** **.** **003**	3.889	1.463–10.335	**0** **.** **006**
Age (per 1 year)	0.936	0.894–0.979	**0** **.** **004**	0.938	0.886–0.993	**0** **.** **028**
Male sex	3.095	0.712–13.45	0.132			
Smoking	2.353	0.900–6.150	**0** **.** **081**	2.150	0.792–5.841	0.133
Diabetes mellitus	1.673	0.706–3.964	0.243			
Hypertension	0.678	0.256–1.791	0.433			
Previous CABG	1.898	0.693–5.197	0.212			
Atrial fibrillation	2.143	0.493–9.311	0.309			
Pre-existing COPD	1.085	0.144–8.164	0.937			
Congestive heart failure	1.513	0.346–6.622	0.582			
Hemoglobin (per 1 g/dL)	1.210	0.931–1.571	0.154			
Creatinine (per 1 mg/dL)	1.338	0.971–1.844	**0** **.** **075**	1.441	1.077–1.927	**0** **.** **014**
CRP (per 1 mg/dL)	1.005	0.979–1.031	0.712			
ß-blocker usage	1.278	0.170–9.585	0.812			
Insulin usage	1.420	0.327–6.159	0.639			
Anticoagulation	2.143	0.493–9.311	0.309			
ACEi/ARB usage	0.99	0.50–1.96	0.977			
SGLT-2i usage	0.781	0.180–3.388	0.742			
Left ventricular ejection fraction (per 1%)	0.982	0.942–1.024	0.387			
PASP (per 1 mmHg)	0.931	0.792–1.094	0.387			
SYNTAX I score (per 1 unit)	1.013	0.954–1.075	0.674			
Presence of LMCA stent	4.682	1.573–13.93	**0** **.** **006**	7.318	2.247–23.83	**<0** **.** **001**
Lesion severity Stable vs. near-occlusion/CTO	0.634	0.260–1.545	0.316			
Device diameter (per 1 mm)	1.055	0.275–4.048	0.937			
Device length (per 1 mm)	0.974	0.933–1.016	0.222			

CABG,: Coronary artery bypass graft surgery; COPD, Chronic obstructive pulmonary disease; CRP, C-reactive protein; ACEi, Angiotensin converting enzyme inhibitors; ARB, Angiotensin II receptor blocker; SGLT-2i, Sodium-glucose cotransporter-2 inhibitors; PASP, Pulmonary artery systolic pressure; LMCA, Left main coronary artery; CTO, Chronic total occlusion; DCB, Drug-coated balloon, SES: Ultrathin-strut sirolimus-eluting stent.

Bold values indicate statistical significance (*p* < 0.05).

In a propensity score-adjusted Cox regression analysis, treatment with ultrathin-strut SES remained associated with a lower risk of TLR in the non-focal ISR subgroup (HR 0.18, 95% CI 0.05–0.66, *p* = 0.009). In the overall cohort, a similar directional association was observed but did not reach statistical significance (HR 0.49, 95% CI 0.23–1.06, *p* = 0.071). No significant difference was observed in focal ISR lesions (HR 1.22, 95% CI 0.46–3.23, *p* = 0.695). Assessment of Schoenfeld residuals demonstrated no significant violations of the proportional hazards assumption in any Cox regression model used in the study (all *p* > 0.05).

A formal interaction analysis was performed to evaluate whether ISR morphology modified the association between treatment strategy and TLR (Table 6). In the unadjusted Cox model, a significant interaction was observed between treatment modality and ISR morphology (HR 0.19, 95% CI 0.06–0.65, *p* = 0.008). This finding remained significant after adjustment using a propensity score-based Cox model (HR 0.18, 95% CI 0.05–0.69, *p* = 0.012). Neither treatment modality nor ISR morphology alone was independently associated with TLR after propensity score adjustment. These findings indicate that the comparative effectiveness of ultrathin-strut SES vs. DCB differed according to ISR pattern, with the benefit of SES being predominantly observed in patients with non-focal ISR lesions.

**Table 6:** Interaction analysis between ISR morphology and treatment modality for target lesion revascularization.

**Variable**	**Unadjusted HR (95% CI)**	***p*-value**	**Propensity-adjusted HR (95% CI)**	***p*-value**
Treatment (SES vs. DCB)	1.31 (0.58–2.96)	0.520	1.08 (0.44–2.67)	0.865
ISR morphology (non-focal vs. focal)	1.82 (0.87–3.83)	0.113	1.67 (0.73–3.84)	0.227
Treatment × ISR morphology interaction	0.19 (0.06–0.65)	0.008	0.18 (0.05–0.69)	0.012

CI, Confidence interval; DCB, Drug-coated balloon; HR, Hazard ratio; ISR, In-stent restenosis; SES, Ultrathin-strut sirolimus-eluting stent.

## Discussion

The main finding of this study is that ultrathin-strut SES is associated with a lower rate of TLR compared with DCB therapy in patients with non-focal DES-ISR. Rates of MACE were also lower in the ultrathin-strut SES group in this subgroup, primarily driven by a reduction in TLR. Despite the presence of more severe lesion types, such as CTO/near-occlusion, and the use of longer devices in the SES group, the advantage of SES in reducing TLR rates persisted. This benefit was consistently observed in time-to-event analyses and remained significant after multivariable adjustment. In contrast, outcomes were comparable between treatment strategies in patients with focal DES-ISR lesions. These findings highlight the importance of lesion characteristics in guiding treatment selection and suggest that the benefit of ultrathin-strut SES may be limited to more complex, non-focal ISR patterns in real-world practice.

The mechanisms of ISR differ according to the type of stent previously implanted. In bare-metal stents (BMS), neointimal hyperplasia is the predominant mechanism due to the absence of antiproliferative drugs (1, 2). Although neointimal hyperplasia also contributes to DES-ISR, late restenosis in DES is more commonly driven by neo-atherosclerosis and stent-related factors such as under-expansion, fracture, or malapposition (1, 2). In BMS-ISR, DCB therapy appears to be a reasonable treatment strategy, as it enables transfer of an antiproliferative drug into the vessel wall, thereby reducing subsequent neointimal hyperplasia. Indeed, previous DAEDALUS study have demonstrated that DCB treatment provides efficacy comparable to repeat DES implantation in BMS-ISR (14). However, the same study showed lower TLR rates with repeat DES implantation compared with DCB therapy in DES-ISR, highlighting the complexity of DES-ISR and underscoring the importance of individualized device selection (14).

In DES-ISR, the underlying mechanism, clinical characteristics, and lesion type/severity are critical in selecting the optimal treatment strategy (18). Although it may be assumed that repeat DES implantation for DES-ISR carries a higher risk of stent thrombosis, previous studies have not demonstrated significant differences between DCB and DES in terms of stent thrombosis rates. Importantly, contemporary ISR management has increasingly evolved from an angiography-guided to a mechanism-guided approach. Intravascular imaging with IVUS or OCT allows identification of the specific substrate responsible for restenosis, including stent under-expansion, malapposition, stent fracture, neo-atherosclerosis, and calcific neo-intimal proliferation, abnormalities that cannot be reliably characterized by angiography alone (19, 20). In addition, the absence of intravascular imaging may have limited the assessment and optimization of lesion preparation. Because these mechanisms directly influence treatment selection, lesion morphology alone may be insufficient to guide the optimal therapeutic approach. For example, when under-expansion is the primary mechanism of restenosis, lesion preparation with high-pressure, cutting, or scoring balloons followed by DCB angioplasty may be adequate, whereas lesions characterized by diffuse neo-atherosclerosis or other substrate-specific abnormalities may derive greater benefit from repeat DES implantation. Consequently, contemporary practice increasingly favors mechanism-guided rather than solely morphology-guided treatment selection (20). In the present study, treatment decisions were based on angiographic findings without routine IVUS or OCT guidance. Therefore, although we observed superior outcomes with ultrathin-strut SES in non-focal ISR lesions, we cannot determine whether this finding was driven by specific underlying mechanisms of restenosis that may have influenced both device selection and subsequent clinical outcomes. This consideration may be particularly relevant for DCB therapy, as failure to adequately correct stent under-expansion or other mechanical causes of ISR before drug delivery may compromise long-term efficacy. Consequently, some of the excess TLR observed in the DCB group may have been related to unrecognized lesion-specific mechanisms that could not be identified without intravascular imaging. The absence of intravascular imaging thus limits the mechanistic interpretation of our results and precludes identification of the biological or structural substrates responsible for the observed treatment effect. Accordingly, while our findings support the potential value of morphology-guided treatment selection in DES-ISR, they should be interpreted as hypothesis-generating and warrant confirmation in prospective studies incorporating intravascular imaging.

Lesion type and severity are also important considerations when selecting a treatment strategy for DES-ISR. Diffuse ISR lesions are associated with a higher risk of recurrent restenosis and target-lesion revascularization (TLR) (16). Previous studies have provided subgroup analyses comparing focal and non-focal ISR patterns. Registry data indicate that DES implantation is more frequently employed than DCB therapy in non-focal DES-ISR (14). Although many studies have shown comparable overall efficacy between DES implantation and DCB angioplasty in DES-ISR, subgroup analyses of non-focal lesions have demonstrated lower target-lesion failure rates with DES during long-term follow-up (19, 21). In line with these observations, expert consensus reports recommend repeat DES implantation in this setting (22, 23). Consistent with these findings, our real-world study demonstrated more frequent use of ultrathin-strut SES in patients with non-focal DES-ISR. Device length was also greater in the SES group, likely reflecting the treatment of more diffuse and complex lesions. Additionally, clinicians may prefer to cover the entire diseased segment, including areas extending beyond the original stent, when implanting a new stent, whereas DCB therapy may be limited to the in-stent segment. Despite the use of longer devices and more complex lesion characteristics in the ultrathin-strut SES group, TLR and MACE rates were significantly higher in the DCB group among patients with non-focal DES-ISR. These findings further support the preferential use, and potential advantage, of repeat DES implantation over DCB angioplasty in this subgroup.

The younger age observed in the DCB group may reflect a tendency among operators to avoid implantation of additional stent layers in younger patients, thereby minimizing long-term metallic burden and preserving future treatment options. Although the rationale for device selection was not systematically recorded, this consideration may have influenced treatment decisions in routine clinical practice and should be taken into account when interpreting the observed age difference between groups.

Stent type and technology may also influence outcomes in DES-ISR. In the DAEDALUS study (14), the type of DES showed a significant interaction with treatment effect, with better outcomes observed for second-generation DES compared with first-generation devices. Further technological advances have introduced ultrathin-strut SES, with strut thickness <70 μm. These devices are designed to enhance biocompatibility and reduce metallic burden, thereby decreasing inflammatory response and the development of neo-atherosclerosis (19). Previous studies have demonstrated reduced TLR rates with ultrathin-strut SES compared with second-generation DES in *de novo* coronary lesions and small-vessel disease (24, 25). However, most existing evidence on ISR relates to first- and second-generation DES, and data regarding ultrathin-strut SES in ISR remain limited.

In a study by Filippo et al. (19), ultrathin-strut SES were compared with thin-strut DES and DCB in patients with DES-ISR. Ultrathin-strut SES were associated with lower TLR rates compared with DCB, both in the overall population and in patients with diffuse ISR. However, the ultrathin-strut SES group included only 15 patients (6%) with focal ISR, limiting conclusions in that subgroup. Our study expands upon these findings by including patients with focal DES-ISR treated with ultrathin-strut SES and demonstrating comparable outcomes between SES and DCB in this subgroup, while confirming lower TLR rates with SES in non-focal ISR.

The observed benefit of ultrathin-strut SES in non-focal ISR may be explained by several potential mechanistic factors. Non-focal ISR lesions typically represent a greater restenotic burden, longer diseased segments, and more extensive neointimal proliferation than focal ISR, thereby increasing the likelihood of residual disease beyond the angiographically apparent target segment. In such lesions, repeat DES implantation may provide more uniform drug delivery throughout the entire restenotic segment and reduced recoil while simultaneously offering additional radial support and scaffolding of diffuse disease. Furthermore, DES implantation may be more effective in treating edge-related disease propagation and preventing geographic miss, which may be particularly relevant in long or diffuse ISR patterns. Compared with earlier-generation DES, contemporary ultrathin-strut SES platforms also provide a lower metallic burden, improved deliverability, more favorable endothelial healing, and reduced flow disturbance while maintaining adequate radial strength (19). Collectively, these characteristics may contribute to superior suppression of recurrent neointimal proliferation in extensive restenotic segments. In contrast, focal ISR lesions often represent localized disease that can be effectively treated with either DCB angioplasty or repeat DES implantation, potentially explaining the absence of a significant treatment difference in this subgroup. Although these mechanisms remain speculative in the absence of routine intravascular imaging, they provide a biologically plausible framework for the interaction observed between ISR morphology and treatment strategy in the present study.

Adequate lesion preparation remains critical in both BMS-ISR and DES-ISR before DCB or DES treatment. The ISAR-DESIRE 4 randomized trial demonstrated superior angiographic outcomes when scoring balloons were routinely used prior to DCB therapy (26). A variety of devices, including scoring balloons, cutting balloons, intravascular lithotripsy, rotational atherectomy, and excimer laser coronary angioplasty, are available for the management of calcified or rigid lesions (27). The use of ultrathin-strut SES in ISR may raise concerns due to their lower radial strength compared with second-generation DES. However, similar concerns apply to DCB therapy, and with adequate lesion preparation both ultrathin-strut SES and DCB can be applied successfully. Therefore, liberal use of adjunctive devices specifically designed for calcified or rigid lesions should be encouraged.

As secondary findings, the presence of LMCA stenting and worse renal function were identified as independent predictors of TLR. LMCA stenting may represent a surrogate marker of more extensive coronary artery disease and greater metallic burden, both of which may increase the risk of restenosis. Renal dysfunction is also a well-established risk factor for adverse outcomes, including TLR (28).

Interestingly, older age independently predicted lower TLR rates in the non-focal ISR subgroup in our analysis. This finding may, at least in part, reflect differences in clinical decision-making across age groups. Younger patients are more likely to undergo repeat coronary angiography and revascularization, which may increase the detection and treatment of restenosis. In contrast, clinicians may adopt a more conservative approach in older patients, often favoring medical therapy and avoiding repeat invasive procedures due to concerns such as comorbidities and procedural risks, including renal impairment. As a result, TLR rates in older patients may be underestimated. Additionally, the occurrence of DES-ISR at a younger age may indicate a more aggressive underlying coronary artery disease phenotype, which may contribute to higher revascularization rates in this population. The relationship between age and TLR remains controversial in the literature. For instance, Kuna et al. (29), in a large study including 3,511 patients specifically designed to address this question, also reported an association between older age and lower TLR rates, even after accounting for the competing risk of death. In our study, long-term mortality rates were relatively low, suggesting that competing risk alone is unlikely to fully explain this finding, and that differences in clinical practice patterns and disease biology may play a more prominent role.

### Clinical implications

The present findings suggest that ISR morphology should be considered when selecting the optimal treatment strategy for DES-ISR. In focal ISR lesions, where restenosis is typically localized, DCB angioplasty appears to remain a reasonable treatment option, offering the advantage of a “leave nothing behind” strategy while avoiding additional stent layers. In contrast, patients with non-focal ISR may derive greater benefit from repeat implantation of a contemporary ultrathin-strut SES, potentially owing to more effective treatment of extensive restenotic segments and improved long-term lesion durability. Although treatment decisions should ideally be guided by intravascular imaging whenever available, our results support an angiography-based approach in routine clinical practice, whereby lesion phenotype may help inform individualized device selection for DES-ISR. A proposed treatment algorithm is presented in the Supplementary Figure S1.

## Conclusion

In this real-world study of patients with DES-ISR, ultrathin-strut SES was associated with lower rates of TLR and MACE than DCB therapy in non-focal lesions, whereas outcomes were comparable between treatment strategies in focal ISR. Formal interaction analysis further suggested that ISR morphology may substantially influence the comparative efficacy of repeat DES implantation and DCB angioplasty. Given the retrospective design and absence of routine intravascular imaging, these findings should be considered hypothesis-generating. Nevertheless, they support the concept of morphology-guided treatment selection in DES-ISR and warrant confirmation in prospective randomized studies incorporating routine intravascular imaging.

## Limitations

This study has several limitations. First, it was a retrospective, single-center observational study, and treatment selection was not randomized; therefore, selection bias and residual confounding cannot be excluded. Second, the sample size was relatively limited, particularly in subgroup analyses. Third, IVUS/OCT was not used, so the underlying mechanisms of ISR could not be systematically assessed. Fourth, follow-up was based on routine clinical practice rather than a standardized angiographic surveillance protocol, which may have influenced TLR detection. In addition, the findings apply to stable DES-ISR patients treated with a single ultrathin-strut SES platform and may not be generalizable to all ISR populations or device types. Finally, the study period spanned a decade, during which PCI techniques, adjunctive pharmacotherapy, and contemporary approaches to ISR management evolved substantially. Temporal changes in device technology, including both SES and DCB platforms, may have influenced treatment selection and clinical outcomes. In addition, only paclitaxel-coated balloons were evaluated in the present study. Therefore, our findings may not be generalizable to newer DCB technologies, including sirolimus-coated balloons, which may have different efficacy and safety profiles. However, because of the limited sample size and the relatively small number of patients treated with individual device platforms, analyses according to specific DCB or SES generations could not be performed reliably. Therefore, devices were grouped according to their broader therapeutic categories (paclitaxel-eluting DCB and ultrathin-strut SES), precluding a comprehensive assessment of the impact of individual device iterations and treatment era on clinical outcomes. Consequently, residual heterogeneity related to temporal changes in clinical practice and device technology cannot be excluded.

## Data Availability

Data supporting the results of this work can be obtained from the corresponding author upon reasonable request.
